# Endothelial Progenitor Cell Levels and Extent of Post-prandial Lipemic Response

**DOI:** 10.3389/fnut.2022.822131

**Published:** 2022-02-14

**Authors:** Buket Akcan, Asim Örem, Yahya Altinkaynak, Birgül Kural, Cihan Örem, Mehmet Sönmez, Mauro Serafini

**Affiliations:** ^1^Department of Nutrition and Dietetics, Faculty of Health, Ardahan University, Ardahan, Turkey; ^2^Department of Medical Biochemistry, Faculty of Medicine, Karadeniz Technical University, Trabzon, Turkey; ^3^Department of Medical Services and Techniques, Ardahan Vocational School of Health Services, Ardahan University, Ardahan, Turkey; ^4^Department of Cardiology, Faculty of Medicine, Karadeniz Technical University, Trabzon, Turkey; ^5^Department of Hematology, Faculty of Medicine, Karadeniz Technical University, Trabzon, Turkey; ^6^Functional Food and Metabolic Stress Prevention Laboratory, Teramo University, Teramo, Italy

**Keywords:** atherosclerosis, endothelial injury, remnant-like lipoproteins, high fat diet, hyperlipidemia

## Abstract

**Background and Objective:**

Due to the frequency of meal ingestion, individuals spend the majority of the day, ~18 h, in a status of post-prandial (PP) stress. Remnant-like lipoprotein particles (RLPs) are predominant in PP phase playing an important role in the development of atherosclerosis. Endothelial progenitor cells (EPCs) have been suggested to play a role in vessel wall homeostasis and in reducing atherosclerosis. However, there is no information about peripheral blood EPCs number following PP stress. We investigated the association between circulating EPCs levels and extent of PP lipemia in healthy subjects following a high-fat meal.

**Materials and Methods:**

A total of 84 healthy subjects (42 men, 42 women) aged 17–55 years were included in the study. PP lipemic response of subjects was determined by Oral Fat-Loading Test (OFLT). All the subjects were classified on the basis of their plasma TG levels after PP lipemic stressors in categories 1 (low), 2 (moderate), and 3 (high). Circulating EPCs numbers were measured by the flow cytometry method.

**Results:**

There was a significant difference in terms of lipid parameters between men and women: high-density lipoprotein cholesterol (HDL-C) was significantly lower in men than in women (*p* < 0.001). Total cholesterol (TC) (*p* = 0.004), low-density lipoprotein cholesterol (LDL-C) (*p* < 0.001), triglyceride (TG) (*p* < 0.001), and TG-AUC (*p* < 0.001) were significantly higher in men than in women. There was no significant difference between the genders in terms of CD34^+^KDR^+^ and CD34^+^KDR^+^CD133^+^cell number and MMP-9 levels. Vascular endothelial growth factor (VEGF) levels were significantly higher in men than women (*p* = 0.004). TC, LDL-C, and TG were significantly higher in the 3rd category than 1st and 2nd categories (*p* < 0.001) in women. Age, body mass index (BMI), fat rate, TG, TC, and LDL-C were significantly higher in the 3rd category than 1st category (*p* < 0.001, *p* = 0.002, *p* = 0.002, *p* = 0.01, *p* = 0.007, *p* = 0.004; respectively), in men. Circulating numbers of EPCs in men were significantly higher in the PP hyperlipidemia group than in the low TG levels category, independently from age (*p* < 0.05). Circulating EPC levels showed a positive correlation with OFLT response in men (*r* = 0.414, *p* < 0.05). Also, OFLT response showed a strong positive correlation with fasting TG levels (*r* = 0.930, *p* < 0.001). EPC levels in categories of women were not different.

**Conclusion:**

Increased EPCs levels in subjects with different PP hyperlipidemia may be associated with a response to endothelial injury, related to increased atherogenic remnant particles at the PP phase.

## Introduction

Atherosclerotic cardiovascular disease (CVD) is one of the leading causes of death worldwide. Epidemiological studies in recent years have shown that hypertriglyceridemia is an independent risk factor for CVDs. In a meta-analysis of 17 prospective studies involving 46.413 men and 10.864 women, Hokanson and Austin emphasized that subjects with a fasting triglycerides (TGs) level higher than 89 mg/dl had a 14% greater risk for CVDs (1). Studies have shown that hypertriglyceridemia is associated with atherosclerosis, but the independence of the serum TG concentration as a causal factor in promoting CVDs is still an object of scientific debate (2–5). Plasma concentrations of TG after a stressor meal seem to be better indicator with respect to fasting TG for predicting CVD (6, 7). Due to the frequency of meal ingestion, individuals spend the majority of the day, ~18 h, in the post-prandial (PP) phase. Remnant-like lipoprotein particles (RLPs) are predominant in the PP phase and they play an important role in the development of atherosclerosis. Many studies have revealed that TG-rich lipoproteins (TRL), especially chylomicron and very LDL (VLDL) remnants, are atherogenic and that delayed removal of chylomicron remnants from the bloodstream induces PP hyperlipidemia (8–10).

Endothelial dysfunction is known to be an early event in atherosclerosis and an important contributor to the pathogenesis of coronary artery disease. The endothelium can repair itself. Cells with the ability to repair the endothelium have been termed endothelial progenitor cells (EPCs). Studies on the molecular mechanisms of atherosclerosis development have emphasized that EPCs may have a very important role in vessel wall homeostasis and in reducing atherosclerosis. Accordingly, the “response to injury” hypothesis of atherosclerosis has been proposed as “EPC-mediated repair of injury” (11, 12). Hill et al. showed a strong correlation between the number of EPCs in peripheral blood and patients with Framingham risk factor scores (13). Studies conducted in the last decade have shown that repair of damaged endothelium occurs not only with cells in that region, but also with contributions from circulating EPCs. EPCs originate from the bone marrow and are released into the circulation under the influence of growth factors such as vascular endothelial growth factor (VEGF) secreted by stimulants such as tissue ischemia (14–16). Various drugs and factors such as exercise also stimulate the circulating release of EPCs. Circulating EPCs adhere to the damaged area and mature in the damaged endothelial area with the effect of adhesion molecules and cytokines (17–19). The balance between the development of endothelial damage in the area of the damaged atherosclerotic lesion and the endothelial repair process determines the outcome of the atherosclerotic lesion. Lack of EPC number and function is associated with the accelerated development of atherosclerosis in these damaged areas. It is seen that almost all the classical risk factors that cause the development of atherosclerosis have negative effects on the number and function of EPC. Many studies have shown that there is an inverse relationship between advanced age, hypertension, diabetes mellitus, smoking and oxidative stress, and the number and functions of EPC (20).

It is known that the TG-rich remnant lipoproteins responsible for the PP hyperlipidemia, cause endothelial damage leading to atherosclerosis. The idea that remnant lipoproteins could induce atherosclerosis by accelerating EPC aging was first suggested by Pu and Ling (21), and then Liu et al. showed that it reduces the proliferation capacity (22). There is no information in the literature about the peripheral blood EPCs number in PP lipidemia. Since people with PP hypertriglyceridemia spend most of their lives and days with high blood atherogenic remnant lipoprotein particles, it is likely to have adverse effects on EPC synthesis and secretion from the bone marrow, and also other risk factors. As it is known that EPCs play a role in repairing endothelial damage it is also possible that remnant lipoproteins are effective on EPCs, their structure, and function (23, 24).

In this study, we investigated the association between circulating EPCs levels and the extent of PP lipemia in healthy subjects following a high-fat meal.

## Materials and Methods

### Subjects

A total of 84 healthy subjects (42 men, 42 women) aged 17–55 years were included in this study. Health status of the subjects was evaluated by means of detailed medical history, physical examinations, and laboratory tests of blood samples [complete blood cell count (CBC), lipids, lipoproteins, and thyroid function tests including TSH, free-T4, liver, and kidney function tests] at the Karadeniz Technical University Farabi Hospital. Exclusion criteria included smoking status, alcohol or drug abuse, presence of acute and chronic inflammatory diseases, chronic kidney disease, obesity, and endocrine disorders related to lipid and lipoprotein metabolism, such as diabetes mellitus, menopause, estrogen replacement therapy, and thyroid hormone disorders. Subjects performing heavy exercise and those on the herbal medicines were also excluded.

### Study Design

This was an acute-randomized dietary intervention trial. At inclusion, a fasting blood sample was obtained and anthropometric measurements were performed. Body weights (kg), body fat percentages, body mass index (BMI) were obtained using impedance scales (Tanita Body Composition Analyzer, TBF-300, Illinois, USA). BMI was calculated using the formula weight/height^2^ (kg/m^2^). The waist-to-hip ratio was calculated by measuring the circumferences at the waist (midway between the rib cage and iliac crest) and hip (maximal circumference between the iliac crest and thigh region). After this stage, OFLT was administered to the subjects and blood sampling was obtained at 2nd, 4th, and 6th h in PP phase. OFLT was performed according to the Cortes and Patsch methods with some changes (25, 26). Fat meal for OFLT was a toast with cheese and butter and as a drink ayran. Ayran is a traditional Turkish beverage made from yogurt or milk and contains carbohydrates, fat, protein, and calcium. The fat meal was containing 24.1% carbohydrate, 62.5% fat, and 13.4% protein representing a total energy content of 1,100 kcal. Subjects were rested for 30 min before the administration of the fat load and then were consumed a fat meal with ayran. Peripheral blood samples were obtained in sodium EDTA coated, sodium citrate coated, and without anticoagulant gelled tubes. Samples were kept on ice and centrifuged immediately for 15 min at 3,000 rpm at 4°C; then, plasma and serum were stored at −80°C until assayed. Blood samples were obtained from subjects daily for analysis of the numbers of EPCs.

The area under the curve (AUC), from fasting TG levels and at 2, 4, and 6 h from OFLT, was obtained value was calculated with the following formula according to the trapezoidal rule. TG-AUC = TG fasting (mg/dl) + 2 × [TG PP 2nd h. (mg/dl) + TG PP 4th h. (mg/dl)] + TG 4th h. (mg/dl).

The study protocol was approved by the local research ethics committee of the Karadeniz Technical University Farabi Hospital (file number: 2010/72-13). All the participants gave written informed consent. The study was conducted according to the recommendations of the Declaration of Helsinki.

### Biochemical Determinations

#### Lipid Analysis

Serum lipid levels were measured enzymatically. TC, HDL-C, and LDL-C levels measured in fasting serum samples, Triglyceride (TG) levels measured in fasting and PP 2nd, 4th, and 6th h serum samples by enzymatic colorimetric method with auto analyzer (Roche Cobas 8000, Modular Clinical Chemistry, GmbH, Mannheim, Germany).

#### Determination of Circulating EPCs by Flow Cytometry

**Figure 1:**
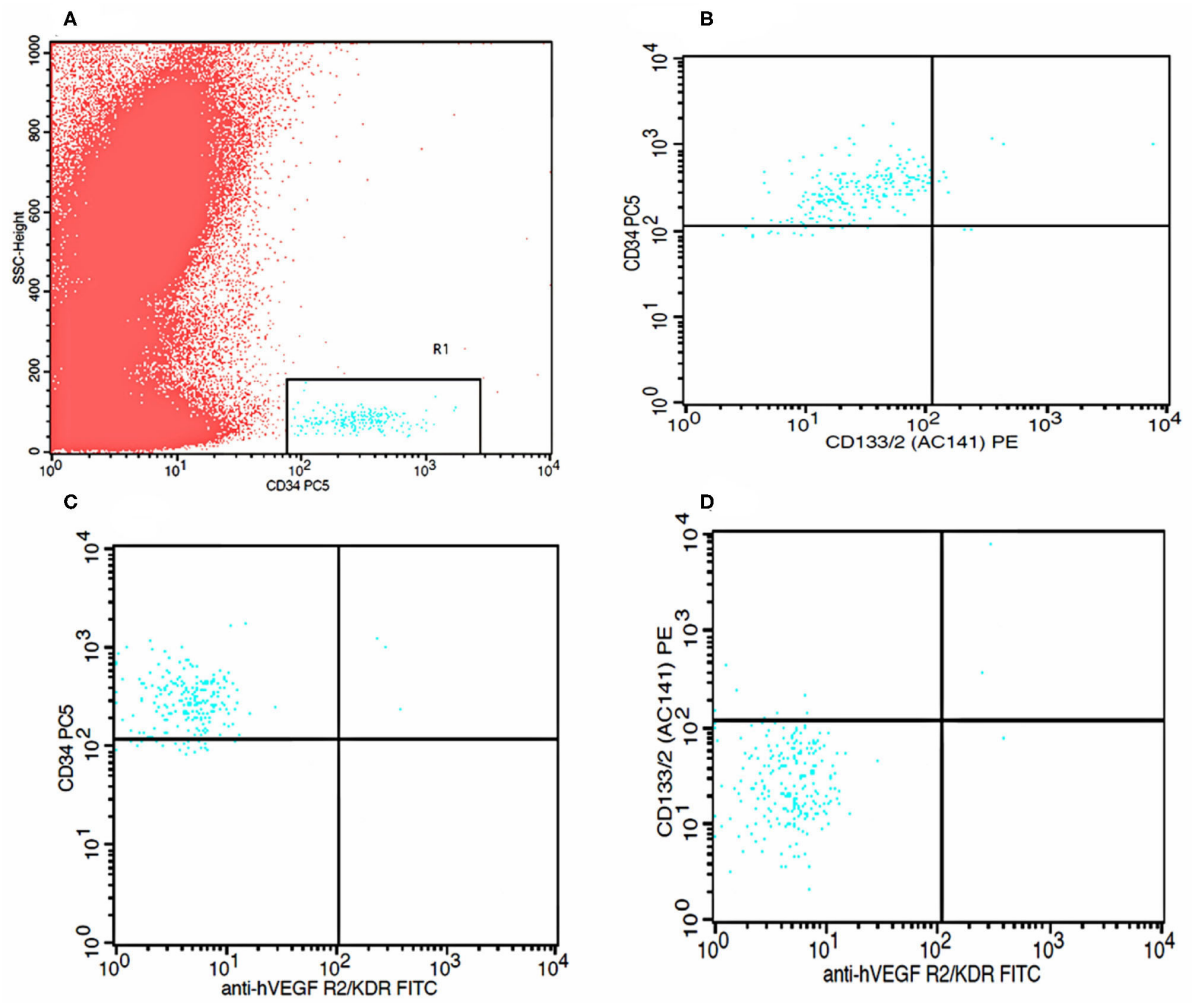
Flow cytometry analysis graphs. **(A)** Identification of CD34 positive cells. **(B)** Identification of CD 133 positive cells within CD34 positive cells. **(C)** Identification of KDR positive cells within CD34 positive cells. **(D)** Identification of both CD133 positive and KDR positive cells within CD34 positive cells.

The number of EPCs was determined by flow cytometry (FACS Calibur, Becton Dickinson, Heidelberg, Germany); briefly, 100 μl whole blood in cubated with fluorochrome-labeled antibodies to CD133 mAB-PE (Miltenyi Biotec, Bergisch Gladbach, Germany), CD34 mAB-PC (Beckman Coulter, Indianapolis, IN, USA), and VEGFR2/KDR-FITC (R&D System, Minneapolis, MN, USA) antibodies at room temperature for 10 minutes. After red cell lysis, the samples were washed and incubated with FITC and PE at room temperature for 15 min. For analysis, first CD34 or CD133 positive cells were gated, identified as a distinct cell population with high expression of the antigens mentioned earlier and low-side scatter, and then these cells were assayed for KDR expression in the mononuclear cell fraction (Figure 1). Cells that were CD34^+^KDR^+^ (late EPCs) and CD34^+^CD133^+^KDR^+^ (early EPCs) were considered EPCs. CD34^+^CD133^+^ cells were considered CECs. For all the analyses, 5 × 10^5^ events were collected and scored using an FACS Calibur analyzer (Becton Dickinson). The data were processed using the Macintosh CELL Quest software package (Becton Dickinson), and the cell count was expressed per one million cytometric events.

#### Enzyme-Linked Immunosorbent Assay

Plasma VEGF and MMP-9 levels were measured using commercial ELISA kits according to the standard protocols of the manufacturer (R&D System Quantikine Human VEGF Immunoassay, Lot: 291989, Catalog Number: DVE00 and R&D System Quantikine Human MMP-9 Immunoassay, Lot: 291696, Catalog Number: DMP900 Minneapolis, MN, USA). The appropriate recombinant human VEGF was used for calibration. The sensitivity of the present assay for VEGF was 9 pg/ml and for MMP-9 was 0.156 ng/ml.

### Statistical Analysis

Data were given as mean and SD for normally distributed variables and median (interquartile range) for non-normally distributed variables. The distribution of variables was assessed by the Kolmogorov-Smirnov test. Comparison of two groups was performed using Student's *t*-test or Mann–Whitney *U*-test. Each sex group was categorized into three equal subgroups based on tertiles of TG-AUC values. Participants with low TG-AUC values were enrolled in Category 1, those with intermediate values in Category 2, and those with high values in Category 3. ANOVA and Kruskal–Wallis test were used to compare parameters among tertiles. Tukey's test was performed for *post-hoc* comparisons. Pearson or Spearman correlation analysis was used to assess the relationships between PP lipemia and atherogenic indices including lipids and lipoproteins and also EPC numbers in the light of the skewness of data distribution. *P* < 0.05 was accepted as statistically significant. Statistical procedures were performed on SPSS for Windows version 16.0 (SPSS Incorporation, Chicago, Illinois, USA) software.

## Results

### Baseline Characteristics of The Study Population

Physical characteristics, biochemical, and immunological parameters of the subjects are shown in Table 1. Men were overweight and older respect to women. There was a significant difference in terms of lipid parameters between men and women: HDL-C was significantly lower in men than in women (*p* < 0.001). TC (*p* = 0.004), LDL-C (*p* < 0.001), TG (*p* < 0.001), and TG-AUC (*p* < 0.001) were significantly higher in men than women. There was no significant difference between the genders in terms of CD34^+^KDR^+^ and CD34^+^KDR^+^CD133^+^cell number and MMP-9 levels. VEGF levels were significantly higher in men than women (*p* = 0.004).

**Table 1:** Physical characteristics and biochemical and immunological parameters of the subjects at baseline.

**Parameters**	**Total study group (*n* = 84)**	**Women (*n* = 42)**	**Men (*n* = 42)**	***P***
Age (years)	30.4 ± 11.0	26.5 ± 8.9	34.4 ± 11.6	0.001
BMI (kg/m^2^)	25.7 ± 4.8	24.2 ± 4.8	27.2 ± 4.2	0.002
TG-AUC (mg/dL/hour)	916.7 ± 476.0	676.3 ± 276.7	1157.0 ± 513.7	<0.001
Waist/Hip	0.8 ± 0.09	0.8 ± 0.07	0.9 ± 0.06	<0.001
Fat rate (%)	24.1 ± 8.2	26.9 ± 8.8	21.2 ± 6.3	0.001
TG (mg/dL)	95.5 ± 51.2	72.8 ±27.9	118.1 ± 58.9	<0.001
TC (mg/dL)	186.2 ± 39.4	174.1 ± 27.9	198.3 ± 45.3	0.004
LDL-C (mg/dL)	108.6 ± 34.9	93.0 ± 21.5	124.1 ± 38.8	<0.001
HDL-C (mg/dL)	53.6 ± 13.4	61.4 ± 12.6	45.9 ± 9.1	<0.001
CD34^+^KDR^+^	2 (1–3)	2 (1–3)	1.5 (1–4)	0.634^*^
CD34^+^KDR^+^ CD133^+^	2 (0–3)	2 (0–3)	1 (0–4)	0.943^*^
VEGF (pg/ml)	55.4 ± 22.4	48.5 ± 21.1	62.3 ± 21.9	0.004
MMP-9 (ng/ml)	397.4 ± 142.8	377.9 ± 118.5	416.8 ± 162.6	0.214

*P-values according to the Student's t-test. Data were expressed as mean ± SD*.

* *P-values according to the Mann–Whitney U-test. Data were expressed as median (interquartile range for 25–75%)*.

*BMI, body mass index; AUC, area under the curve; CD34^+^KDR^+^, late endothelial progenitor cell; CD34^+^KDR^+^CD133^+^, early endothelial progenitor cell*.

### Stratification by Tertiles of TG-AUC Levels

Because TG-AUC levels were found significantly different between genders, women and men were divided separately into tertiles according to their TG-AUC levels and all outcomes were evaluated accordingly. Participants with low TG-AUC values were enrolled in Category 1, those with intermediate values in Category 2, and those with high values in Category 3 as described in Table 2.

**Table 2:** Physical characteristics and biochemical and immunological parameters in women according to the three categories of TG-AUC (*n* = 14), mean ± SD [min–max].

**Parameters TG-AUC (mg/dL/hour)**	**Category 1** **low** **431.1 ±51.2** **[324-523]**	**Category 2** **intermediate** **618.3 ±53.5** **[528-694]**	**Category 3** **high** **979.4 ±262.2** **[722-1639]**	***P***
Age (years)	24.8 ± 7.2	25.4 ± 9.2	29.2 ± 10.22	0.266
BMI (kg/m^2^)	22.9 ± 3.4	24.2 ± 4.8	25.4 ± 5.9	0.403
Waist/Hip	0.75 ± 0.07	0.8 ± 0.06	0.8 ± 0.07	0.369
Fat rate (%)	25.4 ± 8.4	26.9 ± 8.3	28.5 ± 10.1	0.660
TG (mg/dL)	50.5 ± 8.3	69.8 ± 19.3^a^	98.1 ± 28.0^a^^,^^b^	<0.001
TC (mg/dL)	166.3 ± 25.2	158.0 ± 22.9	197.9 ± 18.8^a^^,^^b^	<0.001
LDL-C (mg/dL)	85.4 ± 14.3	82.7 ± 20.4	111.0 ±17.7^a^^,^^b^	<0.001
HDL-C (mg/dL)	66.1 ± 15.2	55.4 ± 8.3	62.6 ± 11.6	0.066
CD34^+^KDR^+^	1.5 (0–3)	2.5 (2–4)	2 (1–3)	0.155^*^
CD34^+^KDR^+^ CD133^+^	1 (0–2)	2 (0–3)	2 (1–3)	0.116^*^
VEGF (pg/ml)	57.5 ± 24.3	44.5 ± 14.3	43.4 ± 21.8	0.146
MMP-9 (ng/ml)	388.3 ± 162.4	406.4 ± 102.9	338.9 ± 68.3	0.304

*P-values according to one-way ANOVA test, post-hoc Tukey's test. Data were expressed as mean ± SD*.

* *P-values according to the Kruskal–Wallis test. Data were expressed as median (interquartile range for 25–75%)*.

a *Significant with respect to category **1***.

b *Significant with respect to category **2***.

*BMI, body mass index; AUC, area under the curve; CD34^+^KDR^+^, late endothelial progenitor cell; CD34^+^KDR^+^CD133^+^, early endothelial progenitor cell*.

There were no significant differences in terms of anthropometric values between the women's categories (Table 2). TC, LDL-C, and TG were significantly higher in the 3^rd^ category than 1^st^ and 2^nd^ categories (*p* < 0.001). TG was significantly higher in the 2^nd^ category than 1^st^ category (*p* < 0.001). Although HDL-C was higher in the 1^st^ category than other categories, no statistically significant differences were found between the categories. There were no significant differences between the categories in terms of CD34^+^KDR^+^ and CD34^+^KDR^+^CD133^+^cell number, VEGF, and MMP- 9 levels (Table 2).

**Figure 2:**
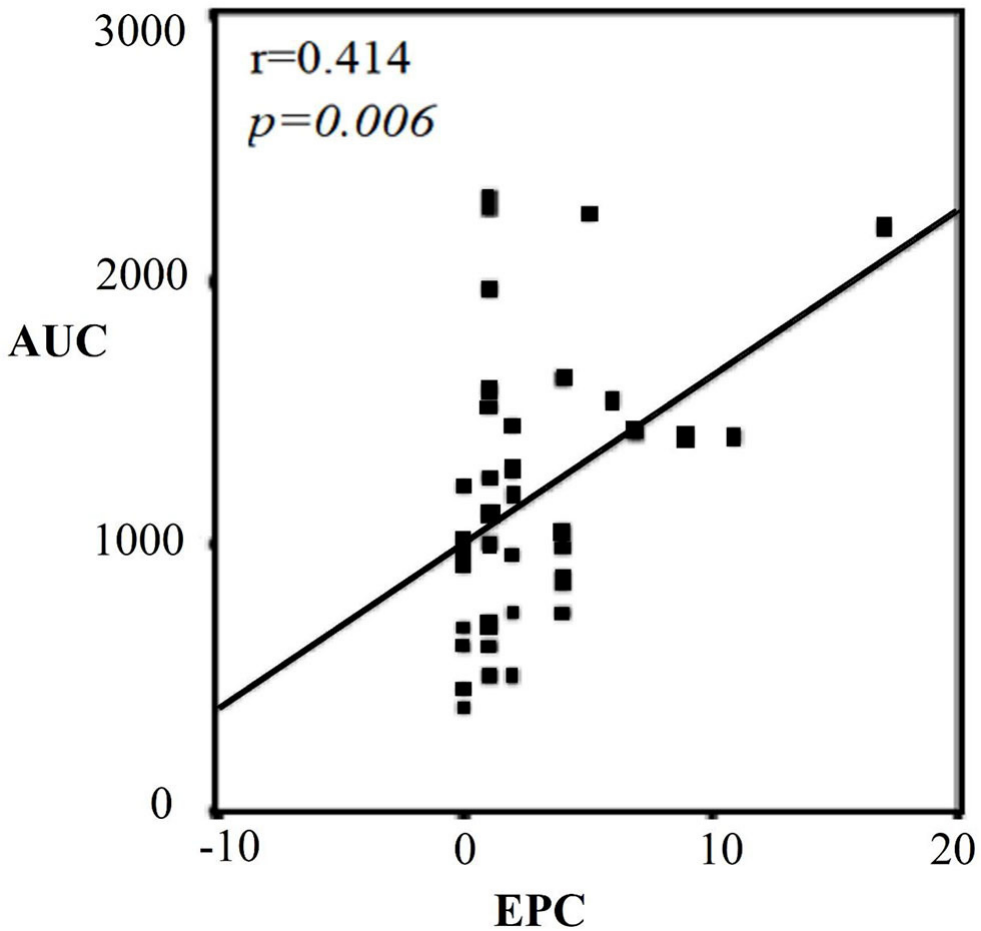
Correlation graph between TG-AUC and CD34^+^KDR^+^CD133^+^ (EPC) in men. Strong correlation between TG-AUC and early EPC numbers (*p* = 0.006). AUC, area under the curve; EPC, endothelial progenitor cell.

There was a significant difference in terms of anthropometric values between the categories of men (Table 3). According to this, age, BMI, and fat rate were higher in the 3^rd^ category than 1^st^ category (*p* = 0.01, *p* = 0.007, *p* = 0.004; respectively). BMI and fat rate were higher in the 2^nd^ category than 1^st^ category. There were no significant differences in terms of waist/hip ratio between the categories. TG, TC, and LDL-C were significantly higher in the 3^rd^ category than 1^st^ category (*p* < 0.001, *p* = 0.002, *p* = 0.002; respectively). HDL-C was significantly lower in the 3^rd^ category than 1^st^ category (*p* = 0.039) LDL-C was highest in the 3^rd^ category and was significantly higher in the 2^nd^ category than 1^st^ category (*p* = 0.002) (Table 3). TG was also significantly higher in the 2^nd^ category than 1^st^ category (*p* < 0.001). CD34^+^KDR^+^ and CD34^+^KDR^+^CD133^+^cell number were significantly higher in the 3^rd^ category than 1^st^ category (*p* = 0.026, *p* = 0.015; respectively). There were no significant differences between the categories in terms of VEGF and MMP-9 levels (Table 3). There was a strong correlation between TG-AUC and early EPC numbers (*p* = 0.006, *r* = 0.414) (Figure 2).

**Table 3:** Physical characteristics and biochemical and immunological parameters in men according to the three categories of TG-AUC (*n* = 14), mean ± SD [min–max].

**Parameters TG-AUC (mg/dL/hour)**	**Category 1** **low** **649.4 ±146.8 [348-887]**	**Category 2** **intermediate** **1068.7 ±120.3** **[926-1279]**	**Category 3** **high** **1752.9 ±357.7** **[1406-2313]**	***P***
Age (years)	27.6 ± 10.9	35.0 ± 11.1	40 ± 9.6^a^	0.01
BMI (kg/m^2^)	24.5 ± 3.7	28.4 ± 4.1^a^	28.9 ± 3.5^a^	0.007
Waist/Hip	0.88 ± 0.07	0.9 ± 0.05	0.9 ± 0.06	0.090
Fat rate (%)	16.8 ± 6.04	27.7 ± 5.7^a^	24.1 ± 5.0^a^	0.004
TG (mg/dL)	68.6 ± 16.5	106.4 ± 25.9^a^	179.4 ± 56.2^a^^,^^b^	<0.001
TC (mg/dL)	168.8 ± 29.6	200.6 ± 25.3	225.4 ± 56.9^a^	0.002
LDL-C (mg/dL)	97.1 ± 26.4	129.1 ±21.4^a^	146.1 ± 47.7^a^	0.002
HDL-C (mg/dL)	50.6 ± 7.5	44.9 ± 5.7	42.2± 11.5^a^	0.039
CD34^+^KDR^+^	1 (0–3)	1 (0–2)	3 (1–7)^a^^,^^b^	0.026^*^
CD34^+^KDR^+^ CD133^+^	1 (0–2)	1 (0–2)	3 (1–7)^a^^,^^b^	0.015^*^
VEGF (pg/ml)	61.7 ± 23.8	61.8 ± 24.5	63.3 ± 18.3	0.980
MMP-9 (ng/ml)	423.5 ± 125.7	481.5 ± 199.9	345.4 ± 132.6	0.082

*P-values according to one-way ANOVA test, post-hoc Tukey's test. Data were expressed as mean ± SD*.

* *P-values according to the Kruskal–Wallis test. Data were expressed as median (interquartile range for 25–75%)*.

a *Significant with respect to category **1***.

b *Significant with respect to category **2***.

*BMI, body mass index; AUC, area under the curve; CD34^+^KDR^+^, late endothelial progenitor cell; CD34^+^KDR^+^CD133^+^, early endothelial progenitor cell*.

### Effects of Age Differences

Women and men were divided separately into two groups according to their ages. BMI, waist/hip ratio, fate rate, and LDL-C were significantly higher in the middle age than the young group in women (*p* = 0.016, *p* < 0.001, *p* = 0.018, *p* = 0.034; respectively) (Table 4). There were no statistically significant differences in terms of TG-AUC, TG, TC, HDL-C, CD34^+^KDR^+^, and CD34^+^KDR^+^CD133^+^ cell number, MMP-9 and VEGF between the young and middle age groups.

**Table 4:** Physical characteristics and biochemical and immunological parameters according to the age in women.

**Parameters**	**Young (*n* = 27)**	**Middle age (*n* = 15)**	***P***
Age (years)	20.9 ± 3.6	36.4 ± 6.8	<0.001
BMI (kg/m^2^)	22.8 ± 4.5	26.5 ± 4.8	0.016
TG-AUC (mg/dL/hour)	627.6 ± 176.8	763.9 ± 391.7	0.128
Waist/Hip	0.7 ± 0.05	0.8 ± 0.06	<0.001
Fat rate (%)	24.6 ± 7.8	31.1 ± 9.3	0.018
TG (mg/dL)	72.6 ± 18.9	73.2 ± 40.2	0.944
TC (mg/dL)	168.7 ± 27.7	183.6 ± 26.8	0.098
LDL-C (mg/dL)	87.8 ± 19.7	102.4 ± 22.1	0.034
HDL-C (mg/dL)	61.6 ± 11.9	60.9 ± 14.2	0.866
CD34^+^KDR^+^	2 (1–4)	2 (1–3)	0.229^*^
CD34^+^KDR^+^CD133^+^	2 (1–3)	2 (0–3)	0.637^*^
VEGF (pg/ml)	49.1 ± 23.1	47.4 ± 17.7	0.809
MMP-9 (ng/ml)	382.1 ± 90.7	370.3 ± 160.4	0.760

*P-values according to the Student's t-test. Data were expressed as mean ± SD*.

* *P-values according to the Mann–Whitney U-test. Data were expressed as median (interquartile range for 25–75%)*.

*BMI, body mass index; AUC, area under the curve; CD34^+^KDR^+^, late endothelial progenitor cell; CD34^+^KDR^+^CD133^+^, early endothelial progenitor cell*.

Body mass index, TG-AUC, waist/hip ratio, fate rate, TG, TC, and LDL-C were significantly higher in the middle age than the young group (*p* = 0.007, *p* = 0.001, *p* = 0.002, *p* = 0.001; *p* = 0.013, *p* = 0.001, *p* < 0.001; respectively), in men (Table 5). There were no statistically significant differences in terms of HDL-C, CD34^+^KDR^+^ and CD34^+^KDR^+^CD133^+^ cell number, MMP-9 and VEGF between the age groups in men.

**Table 5:** Physical characteristics and biochemical and immunological parameters according to the age in men.

**Parameters**	**Young (*n* = 19)**	**Middle age (*n* = 23)**	***P***
Age (years)	24.3 ± 3.8	42.8 ± 8.8	<0.001
BMI (kg/m^2^)	25.4 ± 4.4	28.8 ± 3.4	0.007
TG-AUC (mg/dL/hour)	880.6 ± 410.1	1385.3 ± 483.4	0.001
Waist/Hip	0.9 ± 0.07	0.9 ± 0.05	0.002
Fat rate (%)	17.7 ± 6.6	24.1 ± 4.5	0.001
TG (mg/dL)	98.1 ± 56.1	134.7 ± 57.0	0.043
TC (mg/dL)	173.4 ± 29.2	218.7 ± 46.5	0.001
LDL-C (mg/dL)	102.3 ± 26.5	142.0 ± 38.6	<0.001
HDL-C (mg/dL)	47.0 ± 7.7	45.0 ± 10.2	0.486
CD34^+^KDR^+^	1 (0–2)	2 (1–5)	0.076^*^
CD34^+^KDR^+^CD133^+^	1 (0–2)	1 (1–5)	0.119^*^
VEGF (pg/ml)	63.9 ± 25.1	60.9 ± 19.3	0.660
MMP-9 (ng/ml)	461 ± 134	380.0 ± 177.1	0.099

*P-values according to the Student's t-test. Data were expressed as mean ± SD*.

* *P-values according to the Mann–Whitney U-test. Data were expressed as median (interquartile range for 25–75%)*.

*BMI, body mass index; AUC, area under the curve; CD34^+^KDR^+^, late endothelial progenitor cell; CD34^+^KDR^+^CD133^+^, early endothelial progenitor cell*.

## Discussion

This study showed that PP hypertriglyceridemia affects the number of EPCs *via* remnant-like particles in humans following a dietary stressor. Epidemiological studies in recent years have revealed that hypertriglyceridemia is an individual risk factor for CVDs, independent of the other factors (27–30). Since Zilversmit suggested chylomicrons and chylomicron remnants might play a role in atherogenesis (31), many researchers have conducted studies showing the relationship between PP hyperlipidemia and atherosclerotic diseases, especially CVD. It has been shown that remnant lipoprotein particles initiate early atherosclerosis, have adverse effects on endothelial functions, are associated with atherogenic small dense LDL, and also with prothrombotic and proinflammatory biomarkers such as Factor VII, PAI-1, and CRP (31). Because of the frequency of meal ingestion, individuals spend approximately 18 h in the PP high TG levels. Moreover, the increase in TG levels following a high fat meal has been shown to be associated with an increase of circulating proinflammatory cytokines (32, 33).

When the relationship between PP lipemia and lipids and lipoproteins was examined, it was seen that the parameters were higher in men than women. For this reason, these parameters were evaluated separately in the gender categories. Lipid and lipoprotein profiles in the third category exhibited an atherogenic tendency, especially in male subjects. Many studies such as Women's Health Study, Norwegian Counties Study, and Copenhagen City Heart Study have shown that there is a strong relationship between cardiovascular events and PP TG levels (4, 7, 34). In this study, a strong positive correlation between fasting TG levels and TG-AUC values indicates that the PP lipemic response of individuals with high fasting TG levels may also be impaired. In a study by Wojczynski et al., normal and hypertriglyceridemic female and male individuals were compared and LDL-C was found to be significantly higher in hypertriglyceridemic individuals compared to normotriglyceridemic individuals, while HDL-C was found to be significantly lower (35). There was a very strong positive correlation between fasting TG level and TG-AUC values in both sexes in our study. This supports the idea that people with high-fasting TG levels may also have impaired PP lipemic responses. In the study of Kolovou et al., it was found that each 1 mg/dl increase in fasting TG level caused an increase in TG-AUC value by 8.462 mg/dl/h, partially supporting findings from our study (36). Remnant lipoproteins are risk factors for endothelial dysfunction and therefore atherosclerosis, and EPCs have a crucial role in vessel wall homeostasis and reduction of atherosclerosis (37–39). According to the “EPC-mediated injury repair” hypothesis (11), we aimed to reveal the relationship between PP lipemia and the number of circulating EPCs.

In this study, we showed for the first time the number of EPCs in PP phase. In the literature, there is no *in-vivo* study on relationship between PP lipemia and EPCs. However, Ferreira et al. showed that PP hyperlipidemia increases the circulating levels of endothelial microparticles (EMPs) (40). In this study, low-fat and high-fat diets were applied to 18 subjects in total. Lipid and EMP levels were measured before and 1 and 3 h after the meal. By the flow cytometric analysis, it was observed that high-fat meal significantly increased EMP levels after 1 and 3 h (40). In addition, Liu L. et al. put forward the idea that remnant-like particles may cause atherosclerosis by aging EPCs in 2007, and they published an *in vitro* study in 2009 (21, 22). It was first noted that remnant-like particles can reduce the adhesion, migration and proliferation capacity of EPCs (22). By the biochemical analyzes, it was observed that the EPCs were aged after treatment with remnant lipoproteins (22). In our study, no statistically significant difference was found in terms of EPC numbers between males and females. There was no statistically significant difference between the category of women. In women, lower and closer PP TG levels, higher HDL-C levels, besides the fact that men have a higher risk of atherosclerosis might explain this situation. Studies have shown that HDL increases the number and activity of EPCs (41–43). Various studies have shown that circulating EPCs are reduced in the presence of classic CVD risk factors (44), non-modifiable risk factors such as age and gender also affect the number of EPCs (45). Accordingly, it has been determined that older people have fewer CD34^+^KDR^+^ cells than younger people and men have fewer CD34^+^KDR^+^ cells than women. The number of EPCs may be a factor in explaining the protection of fertile women from CVD risk (45). It can be claimed that the risk of CVD is higher in people with a low EPC count because these people have impaired endothelial repair and compensatory angiogenesis. Hill et al. are the first group to demonstrate a direct correlation between EPC and endothelial function. In their study, endothelial function was evaluated by flow-mediated dilatation technique (13). Later studies also support this correlation (46–48). In this study, EPC levels of men in the high TG-AUC category were found to be significantly higher than in the intermediate and low TG-AUC category. In the high TG-AUC category where the PP TG level is high, the remnant particles remain in circulation for a longer period of time, therefore, susceptibility to atherosclerosis is higher in this category. Based on the literature, it is expected that the number of EPCs in this category, which has a high-atherosclerosis susceptibility, is lower than the other groups. In this case, this result we found is different from the literature. However, it has been demonstrated by various studies that the number of EPCs in the circulation increases in acute events such as heart attack and stroke (37, 49, 50). Therefore, starting from this point, the fact that the number of EPCs increased significantly in the high TG-AUC category brings to mind the situation that develops in response to the existing damage. Despite the fact that the inverse ratio between CVD and EPC number is known, Guven et al. obtained the opposite result in their study; it has been shown that there is a continuous increase in the number of EPCs as the severity of CAD increases (51). When we investigated whether age has an effect on the number of EPCs, it was observed that the number of CD34^+^KDR^+^ cells and CD34^+^KDR^+^CD133^+^ cells did not change with age. Therefore, the number of EPCs varied according to the PP lipemic response, independently of the age factor.

Shintani et al., in their study, compared patients with acute MI and the control group in terms of CD34^+^ mononuclear cells (52). Accordingly, it was observed that CD34^+^ cells were significantly higher in the acute MI group and the levels of these cultured cells reached the highest point on the 7^th^ day, while there was no change in the control group. In this study, besides CD34^+^ cells, plasma VEGF levels were also measured. VEGF, which is one of the factors that allow EPCs to be delivered from the bone marrow to the circulation, was similarly found to be high in patients with acute MI and reached its highest plasma level on the 7^th^ day. VEGF and MMP-9 levels were also evaluated in our study. According to this while MMP-9 levels are not different between males and females, VEGF levels are higher in males than females and were statistically significant. When the categories of women and men were examined, there was no statistically significant difference between the categories in terms of these parameters. Although VEGF levels in the high TG-AUC category in men were higher than the other two categories, consistent with the EPC numbers, this difference in VEGF levels was not statistically significant.

This study has some limitations: it is an acute, 1-day intervention study providing preliminary evidence requiring confirmation in the long-term intervention studies. Moreover, when we stratified the subjects based on age differences, we compared young age (20.9 years) with middle age (36.4 years), without having older subjects that might provide a better piece of knowledge about aging effect. This last aspect will be investigated with a study aim to understand the effect of aging on EPCs.

In conclusion, we showed that PP hypertriglyceridemia affects the number of EPCs *via* remnant-like particles in humans following a dietary stressor and that the highest EPC number was found in the high TG-AUC category. Increased EPCs levels in subjects with PP hyperlipidemia may be associated with a response to endothelial injury related to increased atherogenic remnant particles at the PP phase. Future studies, in a larger number of subjects, are needed to confirm our findings, together with an understanding of the association between EPCs, TG-AUC, and inflammatory response.

## Data Availability Statement

The raw data supporting the conclusions of this article will be made available by the authors, without undue reservation.

## Ethics Statement

The studies involving human participants were reviewed and approved by Ethics Committee of Karadeniz Technical University Farabi Hospital. The patients/participants provided their written informed consent to participate in this study.

## Author Contributions

All authors listed have made a substantial, direct, and intellectual contribution to the work and approved it for publication.

## Funding

This project was funded by the Karadeniz Technical University Research Fund (Project Number: 2010.114.001).

## Conflict of Interest

The authors declare that the research was conducted in the absence of any commercial or financial relationships that could be construed as a potential conflict of interest.

## Publisher's Note

All claims expressed in this article are solely those of the authors and do not necessarily represent those of their affiliated organizations, or those of the publisher, the editors and the reviewers. Any product that may be evaluated in this article, or claim that may be made by its manufacturer, is not guaranteed or endorsed by the publisher.
